# Association of polymorphisms in *TP53* and the promoter region of *IL10* with gastric cancer in a Kazakh population

**DOI:** 10.17305/bjbms.2020.4761

**Published:** 2020-11

**Authors:** Gulmira Kulmambetova, Ivan Shtefanov, Akbota Aitkulova, Meruyert Imanbekova, Aisha Iskakova, Abay Makishev, Yerlan Ramankulov

**Affiliations:** 1 Biotechnology Core Facility, National Center for Biotechnology, Nur-Sultan, Kazakhstan; 2 Department of Oncology, City Oncology Center, Nur-Sultan, Kazakhstan

**Keywords:** Gastric cancer, polymorphism, IL10, TP53, cytokine, SNP

## Abstract

The emerging evidence indicates that single nucleotide polymorphisms (SNPs) of the tumor necrosis factor (*TNF*), interleukin 10 (*IL10*), tumor protein p53 (*TP53*), and cluster of differentiation 14 (*CD14*) genes may determine individual susceptibility to gastric cancer (GC). We aimed to investigate the associations for polymorphisms of the *TNF*, *IL10*, *TP53*, and *CD14* genes in a population of Kazakhs, to identify potential risk or protective associations of the SNPs with GC. A case group of 143 patients hospitalized for GC was enrolled. Controls were 355 volunteers with no history of any cancer and frequency matched with cases by age. Differences in proportions for categorical variables and the assessment of genotypic frequencies conforming to the Hardy–Weinberg equilibrium law were evaluated by the Chi-square test. Associations between genetic polymorphisms and the risk of GC were estimated by regression analysis. For genetic analysis, three genetic models (additive, dominant, and recessive) were used. Four significant associations were found. The SNPs rs1042522 of *TP53* and rs1800896 of *IL10* were risk factors for GC by the additive model. Two polymorphisms of *IL10* were protective of GC, namely, rs1800872 by additive model and rs1800871 by recessive model. No significant associations were observed between the *TNF* and *CD14* polymorphisms and GC. The polymorphisms *TP53* rs1042522 and *IL10* rs1800896 are associated with GC risk, while the polymorphisms *IL10* rs1800872 and rs1800871 are protective of GC in the population of Kazakhs.

## INTRODUCTION

Stomach (gastric) cancer is the fifth most common cancer worldwide, with 1,033,701 new cases diagnosed in 2018, which represents 6.1% of all cancers (www.wcrf.org). The highest incidence of gastric cancer (GC) is in Asia and Latin America; the lowest incidence is in Africa and North America (www.wcrf.org). Cancer is the second most common cause of mortality in Kazakhstan (http://www.medinfo.kz/#/stats). According to data from the Kazakh Research Institute of Oncology and Radiology, mortality from GC in 2017 year was 9.5 per 100,000 population (http://onco.kz/). In the same report, the incidence of all cancers in Kazakhstan was 178.1 per 100,000 population. The Kazakhstan’s age-standardized rate of GC in 2018 was 15.7 per 100,000, which was outnumbered in Asia only by South Korea, Mongolia, Japan, and China (39.6, 33.1, 27.5, and 20.7 respectively). Approximately 16.8% to 34.2% of GC is detected at an early stage in Kazakhstan [1]. The quality of endoscopy and monitoring of patients with precancerous lesions (atrophy, intestinal metaplasia, and dysplasia) are prerequisites for early detection of cancer. Non-invasive screening is considered most suitable for an asymptomatic population. Epidemiology of GC has a regional feature in Kazakhstan. High rates of GC incidence are noted in the eastern and northern provinces, as well as in the Aral Sea and the Caspian Sea regions where excessive consumption of salty foods is observed. The low incidence rate is reported in the South Kazakhstan, where more fruits and vegetables are consumed [2].

GC has a multifactorial etiology involving environmental factors, host susceptibility, bacterial infection, and bacterial pathogenicity. Acknowledged risk factors for GC include *Helicobacter pylori* gastric infection, a diet low in fruits and vegetables, a diet high in salted, smoked, or preserved foods, tobacco smoking, alcohol, obesity, advanced age, male gender, Epstein-Barr virus, and family history [3,4]. Kazakhstan belongs to the countries with an extremely high prevalence of *H. pylori* infection (76.5%) [5]. *H. pylori* infection produces physiological and histological changes in the stomach mucosa that subsequently may lead to the development of cancer [6]. Moreover, the high incidence of clarithromycin resistance in the locally circulating *H. pylori* isolates has reduced the effectiveness of eradication therapy [7]. Additionally, both alcohol consumption and active tobacco smoking are established risk factors for GC. Tobacco use is widespread throughout Kazakhstan. In 2010, the proportion of current smokers in Kazakhstan was 51.2% in all age groups [8]. Alcohol abuse also remains an issue in Kazakhstan. The national survey has found that almost half (45.5%) of the population consumed alcohol at least once a month [9]. High rates of tobacco and alcohol use may serve as aggravating factors for GC in the high-risk Kazakh population. Meanwhile, host genetic factors are also key determinants for developing cancer [10,11]. Genetic polymorphisms in pro-inflammatory and anti-inflammatory cytokine genes influence individual responses to the carcinogenic process [11,12]. Chronic inflammation has been associated with an increased risk of developing several human cancers, including those of the gastrointestinal tract [13]. Studies around the world have particularly implicated specific single nucleotide polymorphisms (SNPs) of several genes in the development of cancers.

It was previously reported that SNPs of the tumor necrosis factor (*TNF*), interleukin 10 (*IL10*), tumor protein p53 (*TP53*), and cluster of differentiation 14 (*CD14*) genes might determine individual susceptibility to GC. The cytokine genes *TNF* and *IL10*, *CD14* - a gene related to innate immunity, and the tumor suppressor gene *TP53* are multifunctional genes involved in the development and progression of many malignant tumors [14,15]. To date, several case-control studies were conducted to detect the associations between *TNF* -308G/A; *IL10* -592G/T, -819G/A, -1082T/C; *CD14* -260 G/A; *TP53* T/G (Arg72Pro) polymorphisms and GC risk in humans [16-21].

Increasing numbers of studies are identifying associations of SNPs in the *TNF*, *IL10*, *TP53*, and *CD14* genes with susceptibility to GC, but the findings are controversial and appear conflicting. Moreover, most such studies were conducted in West and East Asian populations, such as Chinese and Japanese for the latter. Determining the role of SNPs in the epidemiology and pathogenesis of cancer requires describing their diversity in different populations and establishing associations between different SNPs and GC [22]. To the best of our knowledge, there have been no studies in Kazakhstan on the association between polymorphisms of the above-mentioned genes and the risk of GC. The present study aimed to explore whether SNPs in the above-mentioned genes are associated with the risk of GC. We conducted a case-control study focusing on the six most frequently studied polymorphisms (rs1800629 G/A, rs1800872 G/T, rs1800871 G/A, rs1800896 T/C, rs2569190 G/A, and rs1042522 T/G) and GC risk in Kazakhs residing in the northern region of Kazakhstan (Table 1).

**TABLE 1:**
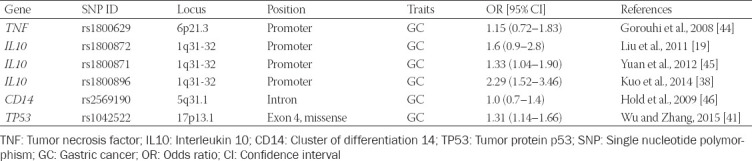
Characteristics of the selected SNPs

## MATERIALS AND METHODS

We conducted a case-control study in a consecutive sample of 143 cases with GC. The controls were 355 healthy volunteers without any history of gastric disorders. The cases were confirmed by pathologic diagnosis at the Oncology Center of Nur-Sultan, in Kazakhstan’s capital. The control group and the study group resided in the same geographical area (the northern Kazakhstan region). Controls were frequency matched to cases by the age group at enrolment [±3 years] (Table 2). Face-to-face interviews collected demographic information (e.g., age and gender) and clinical history, followed by venipuncture to collect a 9 ml blood sample.

**TABLE 2:**
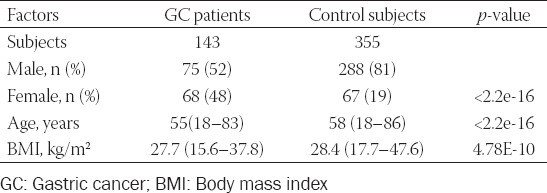
Characteristics of the study population

### DNA extraction and SNP genotyping

DNA was extracted from the venous blood samples using the salting-out method and stored at -70°C until further use [23]. In addition, an in-house paper card-based kit suitable for a collection of human buccal cells was used for the long-term storage of the samples.

We selected six common SNPs in the *TNF, IL10, TP53*, and *CD14* genes based on two criteria. First, these polymorphisms have been reported with a functional effect or statistical association with cancer (http://asia.ensembl.org/Homo_sapiens/Phenotype/Locations). Second, each of these has a minor allele frequency (MAF) of ≥5% in Asian populations, according to the SNP database of the National Centre for Biotechnology Information (www.ncbi.nlm.nih.gov/snp).

SNP genotyping was performed on the Quant Studio 12K Flex (Life Technologies, USA). The total reaction volume was 5 μl, with 2.5 μl of 2x Open Array Real-time master mix and 2.5 μl of DNA (50 ng/μl). Samples were processed according to the standard thermal cycling protocol provided by Life Technologies. Data analysis was performed using the software package TaqMan Genotyper Software v.1.3 (Life Technologies).

### Ethical statement

Before the study, approval was received from the local Ethics Committee of the National Center for Biotechnology (protocol no. 2, 12.03.2012). The Ethics Committee approved the procedures, the informed consent form, and data collected for the study. The investigation was conducted according to the current ethical guidelines. Written informed consent was obtained from all subjects.

### Statistical analysis

Differences in proportions for categorical variables and the assessment of genotypic frequencies conforming to the Hardy–Weinberg equilibrium (HWE) law were evaluated by the Chi-square (*χ*^2^) test (*p* < 0.01 was considered significant). For association studies, we used the z-test or Fisher’s exact test for binomial variables. Associations between genetic polymorphisms and the risk of gastric pathologies were estimated by the unconditional logarithm of logistic regression analysis, producing log odds ratios (OR) and *p*-value. A value of *p* < 0.05 was considered statistically significant. For genetic analysis, three genetic models (additive, dominant, and recessive) were used.

For quantitative non-parametric data, we used the Wilcoxon signed-rank sum test to compare variables between the two groups. At *p* < 0.05 differences were considered statistically significant. Power analysis (with β = 0.20 and α = 0.05) was performed using Power and Sample Size Calculation software [24]. NCBI database was used for comparative analysis of differences in genotype and haplotype frequencies among Kazakh and world populations (www.ncbi.nlm.nih.gov). For analysis of the population, differentiation was used as the exact test. Statistical analysis was performed using R (http://www.R-project.org/).

## RESULTS

Our case-control study enrolled 498 subjects, including 143 cases with GC and 355 controls without a history of GC in the anamnesis. Characteristics of the study population with demographic data are listed in Table 2. The GC group consisted of 75 males and 68 females (median age 55 [range, 18–83] years), median body mass index (BMI) 27.7 [range, 15.6–37.8] kg/m^2^. The control group consisted of 288 males and 67 females (median age 58 [range, 18–86] years), median BMI 28.4 [range, 17.7–47.6] kg/m^2^. The age frequency matching has resulted in slightly, but not significantly higher age in controls than cases (58 vs. 55, respectively). All measured parameters between the cases and control subjects significantly differed.

We genotyped six common SNPs (representing six loci in four genes) and all were in the HWE (Table 3). The distribution fit the HWE law. For this analysis, the major allele homozygotes in all the SNPs were used as the reference genotypes.

**TABLE 3:**
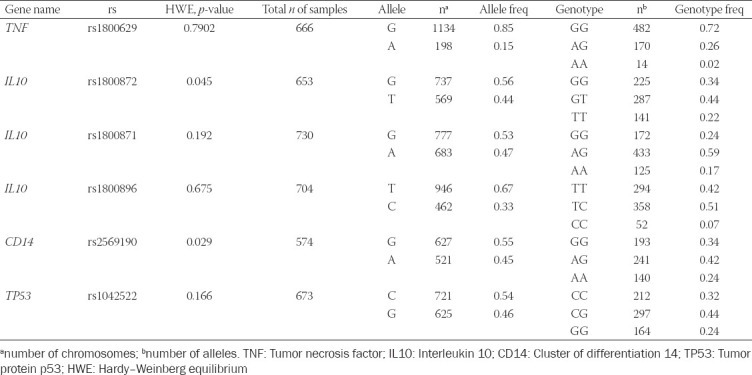
Allele frequency and genotype distribution in the Kazakh population

The allele and genotype frequencies of the six SNPs are summarized in Table 3.

The case-control study for the six polymorphisms was carried out using three genetic models (additive, dominant, and recessive). The results of the logistic regression analysis log (OR) are shown in Table 4. Logistic regression analysis revealed four SNPs inside of four distinct loci that were significantly associated with GC, as follows: rs1800872 (*IL10*), log (OR) = -0.4, *p* = 0.0001 by additive model and log (OR) = -0.7, *p* = 0.0004 by recessive model; rs1800896 (*IL10*), log (OR) = 0.54, *p* = 2.17E-05 by additive model and log (OR) = 0.79, *p* = 3.7E-07 by dominant model; rs1042522 (*TP53*), log (OR) = 0.99, *p* = 3.19E-17 by additive model, log (OR) = 1.05, *p* = 8.04E-10 by dominant model and log (OR) = 1.76, *p* = 3.39E-15 by recessive model; and rs1800871 (*IL10*), log (OR) = -1.10, *p* = 1.06E-07 by recessive model. As a result, the association between phenotype and genotype was tested. In addition, the logistic regression analysis with adjustments for age and gender confirmed the previous results without adjustments (Table 4). No significant associations were found between rs1800629 (*TNF*) and rs2569190 (*CD14*) in our case-control study.

**TABLE 4:**
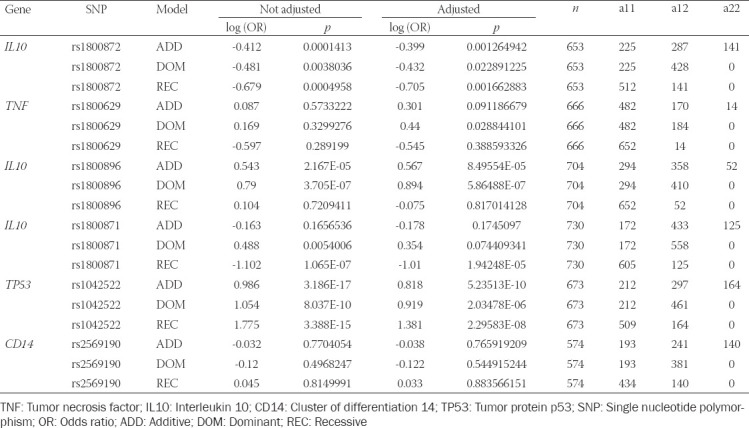
Results of the logistic regression analysis

Additionally, we comparatively analyzed the differences in allele frequencies between the Kazakh population and populations of different ethnic origins represented in the NCBI database, listed as follows: Global; African; East Asian; Europe; South Asian; and American (Table 5).

**TABLE 5:**
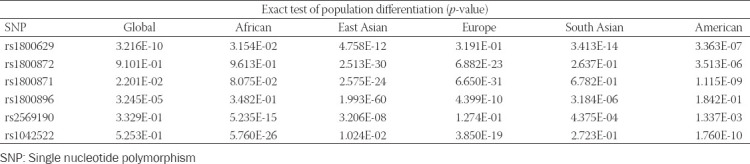
Comparative analysis of allele frequencies between the Kazakhs (present study) and other ethnic populations (NCBI data)

Significant differences in allele frequencies were found between the Kazakhs and other populations for several SNPs. Allele frequencies of five out of six SNPs from the current study were significantly different between the Kazakhs and the East Asian population. Allele frequencies of four SNPs were significantly different among the Kazakhs, European, and American populations. For the South Asian cohort, allele frequencies of three out of six SNPs were significantly different compared to the Kazakh population. Besides, allele frequencies for only two SNPs were significantly different between the African population and the Kazakhs.

## DISCUSSION

Our results support the hypothesis that SNPs are associated with the pathogenesis of GC in the Kazakh population. The proposed model is that polymorphisms involved in susceptibility to gastric disorders may provoke GC [25-27].

According to historical and genetic data, the Kazakhs were formed as a result of the admixture of European and Asian populations [28-30]. A significant proportion of the Caucasoid and Mongoloid components in the Kazakh gene pool is explained by the characteristics of Kazakh ethnogenesis [31]. Anthropologically, the Kazakhs belong to the South Siberian race, formed at the boundary of the Mongoloid and Caucasoid races across the territories of Siberia and Kazakhstan. Archeology and history indicate that the gene pool of Kazakhs was formed as a result of a complex ethnogenetic process involving admixture of the populations from the Near East, East European steppe, East Asia, and Siberia [32,33]. However, according to the comparative genetic analysis, the Kazakh population has its own identity in terms of the studied allelic variants of several genes (*TNF*, *IL10*, *TP53*, and *CD14*). The distribution of allelic variants of the studied SNPs differed significantly among the studied populations (Table 5).

We found that rs1800872 allele T and rs1800871 allele A were directly and significantly protective of GC. We also found that two SNPs were associated with GC, rs1042522 allele G and rs1800896 allele C. The first allele (rs1042522 G) was a risk for GC by three genetic models; the second allele (rs1800896 C) was a risk for GC by additive and dominant models. Therefore, we confirm for the first time these potential genetic markers for GC pathogenesis in a Kazakh population.

Two protective alleles and two risk alleles for GC patients were found in this study. The allele T of rs1800872 [log (OR) = -0.41, *p* = 0.0001 in the additive model and log (OR) = -0.679, *p* = 0.00049 in the recessive model] and the allele A of rs1800871 in *IL10* [log (OR) = -1.1, *p* =1.06E-07 in the recessive model] showed an association with a decreased risk of GC. The other two SNPs, the allele C of rs1800896 in *IL10* [log (OR) = 0.54, *p* = 2.17E-05 in the additive model and log (OR) = 0.79, *p* = 3.7E-07 in the dominant model], the allele G of rs1042522 in *TP53* [log (OR) = 0.98, *p* = 3.19E-17 in the additive model, log (OR) = 1.05, *p* = 8.04E-10 in the dominant model, and log (OR) = 1.77, *p* = 3.39E-15 in the recessive model] showed an association with an increased risk of GC.

The results were matched with several studies suggesting the protective effect of *IL10* in the development of GC [34-36]. It is known that the IL-10 anti-inflammatory cytokines inhibit the production of pro-inflammatory cytokines, thereby reducing the inflammatory response [37]. Besides, the level of *IL10* could also be elevated in inflamed gastric mucosa. However, it should be noted that *IL10*, a powerful pleiotropic cytokine, has the capability of either immunosuppressing or immunostimulating anticancer properties. Some studies show that the *IL10* rs1800871 -819 TT genotype is associated with the common diminished GC risk among Asians. Therefore, the *IL10* -819 TT genotype seems to be protective of GC in Asians [37]. According to other studies, the *IL10* rs1800896 -1082 G allele was associated with increased GC risks (OR 1.2, 95% confidence interval [CI] 0.6–3.2, *p* = 0.007, for the -1082 G allele) [19]. Besides, results from a Taiwanese group showed that those who carry the *IL10* A-1082G allele G have a higher risk of developing GC (*p* = 0.0004) [38]. The comparative analysis of the frequencies of SNPs rs1800872 and rs1800871 showed that these SNPs were significantly different between the Kazakh population and the East Asian, European, and American populations.

The frequencies of SNPs oftentimes vary between ethnic groups. In the present study, the allele frequency of *IL10* rs1800872 G was 0.563 among 498 subjects, but significantly higher than that of the East Asians (0.324), and lower than in the European (0.76) and American (0.67) populations (http://www.ncbi.nlm.nih.gov/SNP). The allele frequency of *IL10* rs1800871 A was 0.531 among 498 subjects, but significantly higher than that of the East Asian population (0.324), and lower than in the European (0.76) and American (0.67) populations. The comparative analysis of the frequencies of SNP rs1800896 showed that they were significantly different between the Kazakh population and the East Asian, European, and South Asian populations. The allele frequency of *IL10* rs1800896 A was 0.328 among 498 subjects, but significantly higher compared to the East Asian (0.052) and South Asian (0.24) populations, and lower than that in the European population (0.453).

It is known that the tumor suppressor gene *TP53* plays an important role in the development of cancer. The *TP53* pathway plays a pivotal role in preventing cancer and in moderating the response to cancer therapies [39]. Besides, the *TP53* gene is one of the most commonly mutated genes in different types of cancers. Mutations of *TP53* can lead to the development of cancers through the inability to initiate the appropriate stress responses [40]. In this study, we found that the polymorphisms of *TP53* (rs1042522) are associated with GC risk in the Kazakh population. The same results were obtained in a Chinese Han population with the value of logistic regression analysis (*TP53* rs1042522: OR 1.69, 95% CI 1.27–2.24 for CC vs. GG; and OR 1.51, 95% CI 1.17–1.94 for GC vs. GG) [41]. Moreover, the evidence from a meta-analysis carried out among Asians (Japan, China, and Korea) suggests that the variant *TP53* Arg72Pro contributes to GC risk [42]. In addition, the *TP53* rs1042522 polymorphisms may be an important biomarker of GC susceptibility for Asians [43].

The comparative analysis of the frequencies of SNP rs1042522 G showed a significant difference between the Kazakh population and the African, European, and American populations. The allele frequency of *TP53* rs1042522 G was 0.533 among 498 subjects, but significantly higher than that of the African population (0.331), and lower than in the European (0.715) and American (0.68) populations.

Considering mutant alleles in the GC samples and control samples, OR, the power of our analysis (α = 0.05), was 0.8 in 143 GC cases and 355 controls with adjusted significant log (OR) for all polymorphisms.

For the other two polymorphisms, namely, rs1800629 (*TNF*) and rs2569190 (*CD14*) reliable association with any of the studied groups was not found, which is most likely due to their small sample size.

There are several limitations to our study, in addition to the small sample size. Our work is a clinical-based case-control study. In this way, it is difficult to avoid the sample selection bias, and subjects may not be representative of the general population. Another potential limitation is that all participants were from the northern region of Kazakhstan; our findings may not necessarily generalize to all Kazakhs or for people in other regions of the world. Different alleles may be present in different sub-populations and have different effects on developing GC. Therefore, it does not enable the extrapolation of our results to other populations.

## CONCLUSION

The present study demonstrated that *TP53* and *IL10* polymorphisms are associated with GC risk in the Kazakh population. Although this result does not permit extrapolation to other ethnic groups, it suggests potential genetic modifiers for GC in the Kazakh population.

Overall, our findings provide evidence of markers for GC risk in the Kazakh population and suggest new approaches for the diagnosis, prognosis, and prevention of a significant cause of morbidity and mortality in Kazakhstan, where only a few studies have been previously reported. Further studies of larger scale may validate and refine our findings, ultimately with the goal of identifying patients at higher risk for developing GC or progressing faster with the disease. Such markers may also have implications for prevention. The future research agenda includes gene therapies to eventually prevent or cure cancers on a larger scale. Increased screening for GC may result in earlier detection, better treatment outcomes, and prevention of GC in the first place.
